# O6-Methylguanine-Methyltransferase (MGMT) Promoter Methylation Status in Glioma Stem-Like Cells is Correlated to Temozolomide Sensitivity Under Differentiation-Promoting Conditions

**DOI:** 10.3390/ijms13066983

**Published:** 2012-06-07

**Authors:** Claire Villalva, Ulrich Cortes, Michel Wager, Jean-Marc Tourani, Pierre Rivet, Celine Marquant, Sebastien Martin, Ali G. Turhan, Lucie Karayan-Tapon

**Affiliations:** 1 INSERM U935, University of Poitiers, Poitiers F-86021, France; E-Mails: claire.gregoire@chu-poitiers.fr (C.V.); ulrich.cortes@chu-poitiers.fr (U.C.); ali.turhan@chu-poitiers.fr (A.G.T.); 2 Department of Hematology and Oncology, University Hospital of Poitiers, University of Poitiers, Poitiers F-86021, France; E-Mails: rivet.pierre.chu@gmail.com (P.R.); marquant.celine.chu@gmail.com (C.M.); martin.sebastien.chu@gmail.com (S.M.); 3 Department of Neurosurgery, University Hospital of Poitiers, University of Poitiers, Poitiers F-86021, France; E-Mail: m.wager@chu-poitiers.fr; 4 Department of Medical Oncology, University Hospital of Poitiers, University of Poitiers, Poitiers F-86021, France; E-Mail: jean-marc.tourani@chu-poitiers.fr

**Keywords:** MGMT methylation, glioma stem-like cells, temozolomide, differentiation

## Abstract

Glioblastoma (GBM) is the most malignant type of primary brain tumor with a very poor prognosis. The actual standard protocol of treatment for GBM patients consists of radiotherapy and concomitant temozolomide (TMZ). However, the therapeutic efficacy of this treatment is limited due to tumor recurrence and TMZ resistance. Recently isolated, glioma stem-like cells (GSCs) are thought to represent the population of tumorigenic cells responsible for GBM resistance and recurrence following surgery and chemotherapy. In addition, MGMT (O6-methylguanine-methyltransferase) methylation is considered as one of the principal mechanisms contributing to TMZ sensitivity of GBM. In this study we have isolated GSCs from 10 adult GBM patients and investigated the relationship between MGMT methylation status and Temozolomide (TMZ) sensitivity of these lines grown either in stem-like or differentiation promoting conditions. Sensitivity to TMZ was significantly associated with MGMT methylation status in cells committed to differentiation but not in stem-like cells. In addition, patients harboring highly methylated MGMT promoters had a longer overall survival. These results reveal the importance of the differentiation process when considering the predictive value of MGMT status in GSCs for clinical response to TMZ.

## 1. Introduction

Temozolomide (TMZ)-based chemotherapy is the standard care for patients with glioblastoma multiform (GBM) in addition to surgery and radiotherapy. A large number of studies have shown that cellular response to alkylating agents such as TMZ inversely correlates with the expression of the DNA repair protein O6-methylguanine-methyltransferase (MGMT) [1,2]. MGMT removes alkylating adducts from the O6 position of guanine and protects cells from cytotoxic and mutagenic effects, conferring a resistance of tumor cells to alkylating agent-based chemotherapy [3]. Expression of MGMT was shown to decrease in some patients, which is the result of epigenetical silencing by methylation of the promoter and coding regions of the *MGMT* gene. Different studies have suggested that MGMT promoter methylation or low MGMT protein levels is associated with TMZ sensitivity in GBM tumors [4–7]. More recently, reports of the EORTC and NCIC trial 26981-22981/CE.3 have established the predictive value of low MGMT methylation for benefit from TMZ treatment in patients with glioblastoma [8,9]. However, despite these promising results, there are conflicting studies about the link between MGMT promoter methylation and TMZ sensitivity [10,11], suggesting that additional factors must be considered to predict glioblastoma sensitivity to TMZ. Recent reports have shown that resistance of glioblastomas to therapy could be conferred by a small fraction of cells endowed with stem cell characteristics and tumor-initiating capacity [12–14]. These glioma stem-like cells (GSCs) present in GBM seem to play a key role in chemoradioresistance due to their increased DNA repair capacity. A study conducted by Murat *et al*. focused on gene expression profiles in 80 GBM treated by chemoradiotherapy (TMZ/RT) and provided evidence for a stem cell-related signature associated with resistance to the therapy [15]. Supporting results have shown that differentiation of GSCs strongly impaired their tumorigenicity and resistance to therapy *in vivo* [16,17]. More recently, increased invasiveness of glioblastoma tumors was found to be related to stem cell properties [18]. In line with these observations, in a recent paper Blough *et al.* analyzed the relationship between MGMT status and TMZ sensitivity in GSCs isolated from 20 patients with glioblastoma [19]. Their study revealed varying sensitivities of GSC lines to TMZ with associations between response to TMZ and expression of MGMT transcript and protein, however they failed to find a correlation with *MGMT* methylation status. In the current study we determined the sensitivity to TMZ of GSCs isolated from GBM patients, in stem-like condition and after commitment to differentiation. Finally, we correlated these data with MGMT promoter methylation.

## 2. Results and Discussion

### 2.1. Primary Culture of Glioma Stem-Like Cells in Undifferentiated and Differentiated States

Early reports have linked stemness properties of GSCs to CD133 expression and suggested that tumorigenic cells in GBM were restricted to the CD133^+^ population [20,21]. For this reason CD133 marker has been widely used in the past to define cancer stem cells, notably in human malignant gliomas. However, recent studies revealed that a subpopulation of CD133^−^ cells isolated from GBM could also be tumorigenic and exhibit similar stemness features [22,23], raising some caution about the use of CD133 to reliably evaluate GBM cell stemness. These observations prompted us to include both CD133^+^ and CD133^−^ GSCs in the present study. The methodology for isolation and characterization of these cells has been previously described in details by our laboratory [24]. In addition, tumorigenicity and stemness properties of GBM derived stem cells were assessed by xenograft experiments in nude mice (Supplementary Figures S1 and S2). Cells isolated from 10 primary GBM tumors (Table 1 and Supplementary Table S1) were cultured in serum-free medium (DMEM/F12 or NBE supplemented with EGF and βEGF) for approximately one week and proliferated as non-adherent multi-cellular spheres (neurospheres). These culture conditions enable tumor cells to retain the molecular characteristics of the primary tumor [24]. When kept in specific differentiation media (10% fetal bovine serum), these cells became elongated and adherent to the surface of the culture flask and were able to differentiate into the three main cell lineages found in the central nervous system: neurons, astrocytes and oligodendrocytes as they expressed beta-tubulin, GFAP, S100, and O4. An index of differentiation was calculated as the ratio between GFAP and Nestin mRNA expression in differentiated cells *vs*. neurospheres (Table 1). Although heterogeneity was present within the data, the index obtained for all GBM cell lines were correlated with the differentiated phenotypes observed by confocal microscopy (Supplementary Figure S3).

### 2.2. Temozolomide Sensitivity

Sensitivity to temozolomide was determined for all GSC lines (GBM-1 to -10) as described in Materials and Methods. Dose-response curves were established after 5 days of treatment and IC_50_, IC_20_, and IC_10_ values were determined (Table 2). Under stem-like conditions, all cell lines were affected by TMZ and cytotoxicity varied among the cells with IC_50_ ranging from 367 μM to 1480 μM (median IC_50_ 630 μM). Clonogenic survival assays performed with GBM1 and GBM2 using IC_50_ dose confirmed these results (data not shown). These data corroborate the previous study from Blough *et al*. [19] showing great variations in response to TMZ at clinical doses (below 50 μM). When cultured in differentiation medium, cells exhibited different IC_50_ values (354 μM to 3950 μM) (median IC_50_ 568.5 μM), with one highly resistant line. Spearman’s correlation test showed no relationship between IC_50_ in stem-like condition and after differentiation. The dose-response curves obtained in this experiment indicate that TMZ doses achieved in clinical applications (5–50 μM; 1–10 μg/mL) have a corresponding efficacy below IC_10_ (*i.e.*, 10% of cell death) after 5 days of treatment for both neurospheres and differentiated cells. Interestingly, comparison of the IC_50_ values indicates that TMZ sensitivity of GSCs was slightly increased when cells were cultured in differentiation medium (median IC_50_ 568 μM *vs*. 630 μM), with seven out of 10 lines showing higher sensitivity after commitment. These results contrast with previous work from Beier *et al*. suggesting that temozolomide preferentially depletes the stem cell subpopulation in GBM (CD133^+^ and CD133^−^) but spares more differentiated cells [25]. The most likely explanation for the discrepancy comes from the fact that these authors did not analyse the effect of temozolomide under fully differentiating conditions (FBS-containing medium).

### 2.3. MGMT Promoter Methylation Status in GBM Tumors, Neurospheres and Differentiated Cells

MGMT-methylation status of the tumor is an independant prognostic factor for GBM patients treated with an alkylating agent such as TMZ [7,25]. Moreover, the epigenetic inactivation of the *MGMT* gene is associated with a better response to chemotherapy [4,5]. By using a quantitative pyrosequencing approach [26], we determined the methylation status of the *MGMT* gene promoter region (Table 3). This quantitative methylation assay detects the level of methylation of 5 CpG sites located in the first exon of the *MGMT* gene and is of high prognostic value compared to MS-PCR and qMS-PCR [26]. CpG positions assessed in this pyrosequencing assay overlap the region analyzed by qMS-PCR and represent a stable indicator of the overall methylation level. Table 3 shows the methylation status of each CpG island for the 10 tumoral samples and corresponding GSCs in stem-like or differentiated condition. The median methylation level for all five CpG sites tested was 2% in the tumors, 3.5% in neurospheres and 10% in differentiated cells. Statistical analysis of median CpG methylation levels showed significant positive correlations between stem-like and differentiated cells (*p* = 0.0003), between tumor and stem-like cells (*p* = 0.0007), between tumor and differentiated cells (*p* = 0.0005) (Table 4). These observations indicate that the overall tumor methylation pattern is conserved among GSCs, independently of their differentiation status. Comparison of median CpG levels reveals a higher MGMT promoter methylation when cells were kept in differentiation medium (10% *vs*. 3.5% in stem-like conditions). Nevertheless, statistical analysis between median CpG levels in stem-like conditions *vs*. differentiating conditions did not show significant difference, most likely due to the great variability between cell lines. Further analysis of MGMT transcript expression revealed no significant difference when cells were maintained in either conditions, although highly variable levels were observed between cell lines (data not shown). Interestingly, the methylation status of the MGMT promoter was correlated with the expression levels of MGMT transcript in both stem-like and differentiating conditions (*p* < 0.01 by the Spearman’s correlation test, data not shown).

### 2.4. Relationship between MGMT Promoter Methylation and Sensitivity to Temozolomide

**Figure 1:**
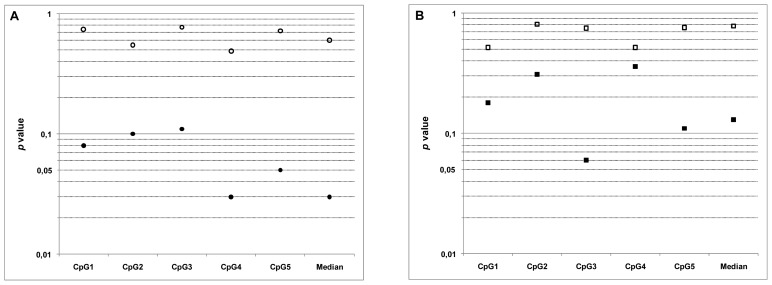
Graphical representation of *p* values from Spearman’s correlation test in Table 5. (**A**) Correlations between TMZ sensitivity and MGMT methylation status in GSCs in stem-like conditions (○) and after differentiation (●); (**B**) Correlations between TMZ sensitivity of GSCs in stem-like conditions (□), after differentiation (■), and MGMT methylation status in corresponding tumors.

Correlations of TMZ sensitivity with MGMT methylation status were analyzed in GSCs in stem-like conditions and after differentiation. As shown in Table 5 and in Figure 1, no correlations were found between IC_50_ of TMZ and MGMT methylation in neurospheres, either at individual CpG site, or with the median CpG value. These observations are in accordance with previous results from Blough *et al*. [19]. However, when cells were kept in differentiation promoting conditions a significant negative correlation was found, despite the reduced number of lines available (*p* = 0.03 with median CpG value). Hence, for the first time our data demonstrate that GSCs engaged in the differentiation process are more sensitive to TMZ and this sensitivity is correlated to the methylation status of MGMT.

In accordance with our results, a recent analysis of gene expression signatures associated with TMZ resistance in malignant glioma also identified MGMT level as a predictor of TMZ response in GBM cells grown under differentiating conditions [27]. Together these results indicate that the differentiation status of GBM cells should be taken into account when considering the predictive value of MGMT methylation in response to TMZ.

Although GBM tumors contain in majority differentiated cells, statistical analysis of the five CpG islands showed no relationship between MGMT promoter methylation in tumors and GSCs sensitivity to TMZ (Table 5 and Figure 1B). Interestingly, the comparison of median *p* values tend to suggest a better predictive value of the methylation status of MGMT in highly differentiated tumors. One possible explanation for the lack of correlation could be due to the cellular heterogeneity of the tumor. To answer this question we determined the differentiation level of the 10 tumors included in this study by calculating the ratio GFAP *vs*. nestin expression (Supplementary Table S2). Results obtained indicate that capacity for differentiation is highly heterogeneous between tumors, with extensive 500-fold variations in differentiation levels. Hence, tumor heterogeneity might be one explanation for the lack of correlation between MGMT status in this set of tumors and TMZ sensitivity of differentiated cells. These data uphold previous immunohistological observations showing intra- and intertumoral heterogeneity in the distribution of anaplastic and differentiated cells in glioblastomas [28]. The methylation status of the MGMT gene promoter is a promising molecular predictive marker, but conflicting studies with controversial data have hampered its clinical use. Our results tend to indicate a major effect of the tumor differentiation status in such studies, which could explain the discrepancies observed in the literature. Further studies on larger cohorts of tumors stratified by their differentiation status are needed to establish the relationship between MGMT promoter methylation and TMZ sensitivity in GBM tumors.

Notwithstanding of the degree of differentiation of the tumors, long-term follow-up of patients in whom GSCs have been isolated revealed a longer overall survival for those with higher MGMT methylation levels. Patients in whom GBM3, 5, 6 and 9 have been isolated had a median survival period of 420 days while other patients had a 306 days median survival. To minimize the risk of selection bias associated with this reduced cohort, all patients included in this study were consecutively treated at our hospital over a 3 years period, were aged-matched and carried wild-type IDH1 and IDH2 genes. Although statistical analysis cannot be performed with a reduced number of patients, these data are in line with previous reports [29] and suggest a prognostic significance for MGMT methylation in GBM tumors.

## 3. Experimental Section

### 3.1. GBM Samples

Tumor samples were obtained from 10 adult GBM patients (GBM1 to GBM10) and processed within 30 min after surgical resection (patients characteristics are summarized in Table 1). These patients were all initially oriented after surgery towards the treatment protocol described by Stupp *et al.* [30]. Written informed consent forms were obtained from each patient. Primary cultures were derived from tumor samples, cultured and characterized as described previously [24].

### 3.2. RT-qPCR

Nestin, GFAP, and MGMT mRNA expression were determined by Taqman gene expression assays (Applied Biosystems, Foster City, USA) as described previously [24] and normalized to GAPDH mRNA levels present in the same cDNA sample. Relative changes in nestin, GFAP and MGMT mRNA amounts were determined by the 2^−ΔΔCt^ method.

### 3.3. Proliferation and Cytotoxicity Assays

Doubling times of cultures and cytotoxicity of Temozolomide (Interchim, Montluçon, France) were determined at passage 10 using the XTT cell proliferation test (Roche, Basel, Switzerland) as previously described [27]. Before analyses, cells from neurospheres were dissociated and seeded in 96- well plates at a density of 5 × 10^4^ cells per well and the quantification of viable cells was performed on a MRXII (Dynex Technologies, Chantilly, VA, USA) at 450 nm. The 50% inhibitory concentrations (IC_50_) of Temozolomide were determined after treatment with increasing amounts for five days.

### 3.4. DNA Extraction and Bisulfitation

DNA was extracted from frozen tumor samples and cells in culture using the Qi-Amp DNA minikit or the DNeasy tissue kit (Qiagen, Hilden, Germany) in accordance to the manufacturer’s instructions. Quantification was performed on a Nanovue spectrophotometer (General Healthcare, Brentford, UK). 500 ng of genomic DNA were bisulfited using the EZ DNA methylation Gold kit according to protocol (Zymo Research, Orange, CA, USA). Universal methylated DNA (Millipore, Billerica, MA, USA) were included as negative and positive controls.

### 3.5. Quantification of Methylation by Pyrosequencing

The pyrosequencing methylation assay was performed with the PyroMark^TM^ MGMT kit (Qiagen, Hilden, Germany) on a Q24 MDx system (Qiagen, Hilden, Germany), according to the manufacturer’s protocol. The PyroMark^TM^ MGMT kit detects the level of methylation of 5 CpG sites located in the first exon (+17 to +39) of the *MGMT* gene. A cytosine not followed by a guanine, and which was therefore not methylated, was used as an internal control for completion of the bisulfite treatment. Universal methylated DNA (Millipore, Watford, UK) and unmethylated DNA were included as control. The mean methylation value of the positive and negative controls were 97% and 3% respectively.

### 3.6. Statistical Analysis

Correlation analyses were done by using the Spearman’s rank correlation test. Statistical significance was determined according to the Spearman’s rank correlation coefficient (rho). For *n* = 10, a rho value > |0.6483| was considered to indicate statistical significance (*p* < 0.05).

## 4. Conclusions

In conclusion, we have isolated 10 GSC lines from adult GBM patients and investigated the relationship between MGMT methylation status and TMZ sensitivity of these lines in stem-like or differentiation promoting conditions. The results indicate that sensitivity to TMZ is correlated to the methylation status of MGMT in GSCs engaged in the differentiation process but not in stem-like cells. Hence the present study could provide a new step for establishing the involvement of MGMT status in glioblastoma, taking into account the importance of the differentiation status of cells in response to TMZ and alkylating agents.

## Supplementary Materials



## Figures and Tables

**Table 1:** Patients’ characteristics and corresponding glioma stem-like cells (GSCs) lines. The index of differentiation was determined after 7 days of culture in FBS-containing medium and was calculated as the ratio between GFAP/nestin in differentiated cells and GFAP/nestin in neurospheres.

Patient #	Sex	Age (Years)	Tumor location	OMS classification	Overall survival (Months)	Cell line	Index of differentitaion
1	M	69	Left fronto-parietal	Grade IV	15	GBM1	83.5
2	M	57	Right fronto-temporal	Grade IV	10	GBM2	31.6
3	M	53	Right parieto-occipital	Grade IV	10	GBM3	6.75
4	M	51	Left frontal	Grade IV	13	GBM4	34.3
5	M	56	Left temporo-frontal	Grade IV	6	GBM5	2.26
6	M	69	Right basi-frontal	Grade IV	More than 18	GBM6	382
7	M	59	Left temporal	Grade IV	10	GBM7	12.4
8	F	65	Right frontal	Grade IV	13	GBM8	4.52
9	M	61	Right temporal	Grade IV	More than 18	GBM9	7.71
10	M	56	Left parieto-occipital	Grade IV	10	GBM10	19.3

**Table 2:** IC_50_, IC_20_, and IC_10_ values for temozolomide (TMZ) effects on GSCs. GSCs were plated in quadruplicate into 96-well plates and maintained in serum-free media (neurospheres) or differentiation media (differentiated cells). After 5 days, viable cells were counted and IC_50,_ IC_20_, and IC_10_ values were determined as described in materials and methods.

Cell line	Neurospheres			Differentiated cells		
	
	IC_10_ (μM)	IC_20_ (μM)	IC_50_ (μM)	IC_10_ (μM)	IC_20_ (μM)	IC_50_ (μM)
GBM1	69	194	516	121	267	493
GBM2	84	194	474	259	503	1236
GBM3	185	346	830	128	239	570
GBM4	133	277	707	60	174	515
GBM5	50	134	367	74	180	354
GBM6	401	908	1480	94	212	567
GBM7	186	311	686	110	227	577
GBM8	181	280	574	283	557	784
GBM9	65	185	495	80	190	413
GBM10	202	349	787	894	1770	3950
Median	157	278.5	630	115.5	233	568.5

**Table 3:** Quantitative O6-methylguanine-methyltransferase (MGMT) promoter methylation analysis. The table shows individual quantification of five CpG sites located in the promoter region of the MGMT gene in all 10 tumors and derived GSCs. Corresponding IC_50_ values for TMZ are indicated.

Cell Line	CpG1			CpG2			CpG3			CpG4			CpG5			Median CpG			NS-IC_50_	DC-IC_50_
					
	NS	DC	Tumor	NS	DC	Tumor	NS	DC	Tumor	NS	DC	Tumor	NS	DC	Tumor	NS	DC	Tumor		
GBM1	3	14	1	7	33	2	4	20	2	5	31	1	3	13	2	4	20	2	516	493
GBM2	1	1	1	2	3	3	2	2	2	1	2	2	2	1	2	2	2	2	474	1236
GBM3	16	11	7	42	28	14	41	22	11	31	18	11	41	18	12	41	18	11	830	570
GBM4	1	1	2	3	2	4	2	1	2	3	3	4	2	2	2	2	2	2	707	515
GBM5	72	76	13	85	88	54	37	56	13	29	52	15	33	49	24	37	56	15	367	354
GBM6	97	96	53	98	97	52	56	84	12	95	99	35	88	94	47	95	96	47	1480	567
GBM7	1	1	1	2	2	2	1	2	2	2	8	2	1	1	2	1	2	2	686	577
GBM8	1	1	1	3	2	3	2	2	2	3	10	3	1	2	1	2	2	2	574	784
GBM9	94	95	10	97	98	29	87	89	28	99	98	35	94	94	25	94	95	28	495	413
GBM10	2	2	2	13	4	5	3	2	2	14	3	7	3	2	2	3	2	2	787	3950
Median	2.5	6.5	2	10	16	4.5	3.5	11	2	9.5	14	5.5	3	7.5	2	3.5	10	2	630	568.5

NS: neurospheres; DC: differentiated cells.

**Table 4:** Spearman’s correlation test on MGMT methylation status in Glioblastoma (GBM) tumors and corresponding GSCs.

	CpG1 *	CpG2 *	CpG3 *	CpG4 *	CpG5 *	Median CpG #
Meth NS/Meth DC	*p* < 0.0001	*p* = 0.0007	*p* = 0.0003	*p* = 0.004	*p* = 0.0003	*p* = 0.0003
	*r* = 0.99	*r* = 0.88	*r* = 0.91	*r* = 0.81	*r* = 0.90	*r* = 0.90

Meth Tum/Meth NS	*p* = 0.002	*p* = 0.002	*p* = 0.002	*p* = 0.002	*p* = 0.0004	*p* = 0.0007
	*r* = 0.85	*r* = 0.85	*r* = 0.86	*r* = 0.86	*r* = 0.90	*r* = 0.88

Meth Tum/Meth DC	*p* = 0.007	*p* = 0.03	*p* = 0.0004	*p* = 0.05	*p* = 0.003	*p* = 0.0005
	*r* = 0.79	*r* = 0.68	*r* = 0.90	*r* = 0.62	*r* = 0.83	*r* = 0.90

The correlation coefficient, Spearman’s rank correlation coefficient (rho, *r*) is given together with *p*, *p* < 0.05 is considered statistically significant;

* Statistical analysis of CpG1, 2, 3, 4 and 5 have been performed without correction factor and are given for indication only;

# Median CpG values have been analyzed in single comparison tests; NS: neurospheres; DC: differentiated cells; Tum: tumor.

**Table 5:** Spearman’s correlation test between sensitivity to TMZ (IC50) and MGMT methylation status in GBM tumors and derived GSCs.

	CpG1 *	CpG2 *	CpG3 *	CpG4 *	CpG5 *	Median CpG #
IC_50_ NS/Meth NS	*p* = 0.74	*p* = 0.55	*p* = 0.77	*p* = 0.49	*p* = 0.72	*p* = 0.60
	*r* = 0.12	*r* = 0.21	*r* = 0.10	*r* = 0.25	*r* = 0.13	*r* = 0.19

IC_50_ DS/Meth DS	*p* = 0.08	*p* = 0.10	*p* = 0.11	***p*** **= 0.03**	*p* = 0.05	***p*** **= 0.03**
	*r* = −0.58	*r* = −0.55	*r* = −0.54	***r*** **= −0.67**	*r* = −0.62	***r*** **= −0.68**

IC_50_ NS/Meth Tum	*p* = 0.52	*p* = 0.81	*p* = 0.75	*p* = 0.52	*p* = 0.76	*p* = 0.78
	*r* = 0.23	*r* = 0.09	*r* = −0.12	*r* = 0.23	*r* = 0.10	*r* = 0.10

IC_50_ DS/Meth Tum	*p* = 0.18	*p* = 0.31	*p* = 0.06	*p* = 0.36	*p* = 0.11	*p* = 0.13
	*r* = −0.46	*r* = −0.36	*r* = −0.61	*r* = −0.32	*r* = −0.54	*r* = −0.51

The correlation coefficient, Spearman’s rank correlation coefficient (rho, *r*) is given together with p, *p* < 0.05 is considered statistically significant;

* Statistical analysis of CpG1, 2, 3, 4 and 5 have been performed without correction factor and are given for indication only;

# Median CpG values have been analyzed in single comparison tests; NS: neurospheres; DC: differentiated cells; Tum: tumor.
