# Supplementary material for: O6-Methylguanine-Methyltransferase (MGMT) Promoter Methylation Status in Glioma Stem-Like Cells is Correlated to Temozolomide Sensitivity Under Differentiation-Promoting Conditions
Source: Int J Mol Sci. 2012 Jun 7;13(6):6983–94. doi: 10.3390/ijms13066983 (PMC3397507; doi:10.3390/ijms13066983)

Supplementary Materials

Table S1: Supplementary informations on patients 1 to 10. KPS, Karnofsky Performance Status Scale; IDH, Isocitrate Dehydrogenase; RT, Radiotherapy; CT, Chemotherapy; TMZ, Temozolomide; BCNU, bis-chloroethylnitrosourea; CA, Camptothecin/Avastin; ND, No data.

Patient #	KPS	Focality	Extent of Resection	Ki67	Necrosis	IDH mutations		Primary/Reccurent Tumor	Treatment		Stupp protocol	Progression-free survival
						IDH1	*IDH2*		RT	CT		
**1**	70	Unifocal, then multifocal at 6 months	Total	30%	Yes	No	No	Primary	60 Gy	TMZ	Yes	8
**2**	70	Unifocal, then multifocal at 9 months	Subtotal	ND	Yes	No	No	Primary	60 Gy	TMZ/BCNU	Yes	3
**3**	80	Unifocal, then multifocal at 4 months	Biopsy	ND	Yes	No	No	Primary	60 Gy	TMZ/BCNU	Yes	2
**4**	70	Unifocal, then multifocal at 5 months	Total	50%	Yes	No	No	Primary	60 Gy	TMZ	Yes	6
**5**	80	Unifocal	Partial	ND	Yes	No	No	Primary	No	TMZ	No (TMZ only)	2
**6**	50	Unifocal	Total	ND	Yes	No	No	Primary	40 Gy	TMZ/CA/BCNU	Yes	10
**7**	100	Unifocal	Biopsy	ND	Yes	No	No	Primary	60 Gy	TMZ	Yes	5
**8**	70	Bifocal	Biopsy	ND	Yes	No	No	Primary	60 Gy	TMZ	Yes	6
**9**	80	Unifocal, then multifocal at 5 months	Total	20%	Yes	No	No	Primary	60 Gy	TMZ	Yes	10
**10**	70	Unifocal	Total	ND	Yes	No	No	Primary	60 Gy	TMZ	Yes	7

Table S2: Differentiation levels in glioblastoma tissues obtained from patients 1 to 10. Differentiation was assessed by the ratio GFAP/nestin in representative samples from each tumor.

Patient #	Tumor differentiation level
1	48,84
2	10,55
3	55,87
4	27,36
5	0,68
6	10,59
7	342,51
8	0,71
9	2,73
10	45,41

Figure S1: *In vitro* and *in vivo* analysis of glioblastoma-derived GSCs. (**A**) Representative images of neurospheres isolated from a primary glioblastoma and grown under stem-like (right) and differentiation (left) conditions; (**B**) Cytologic aberrations and mitotic figures in stem-like cells; (**C**) Detection of immaturity markers in neurospheres (Nestin, Sox2, CD133) and differentiation markers of three neural lineages (GFAP, S100, astroglial; β-tubulin, neuronal; O4, oligodendroglial); (**D**) Neurosphere-derived cells are tumorigenic and initiate tumors in nude mice after orthotopic injection of high (10^5) and low number (10^3) of cells. Hematoxylin and eosin staining were performed on 15-µm thick cryostat sections; (**E**) Neurospheres from glioblastomas show the same genomic abnormalities than respective paired tumors, with loss of heterozygosity on marker D10S541 (GBM1), D9S157 (GBM2), D19S112 (GBM6) and D19S412 (GBM10).

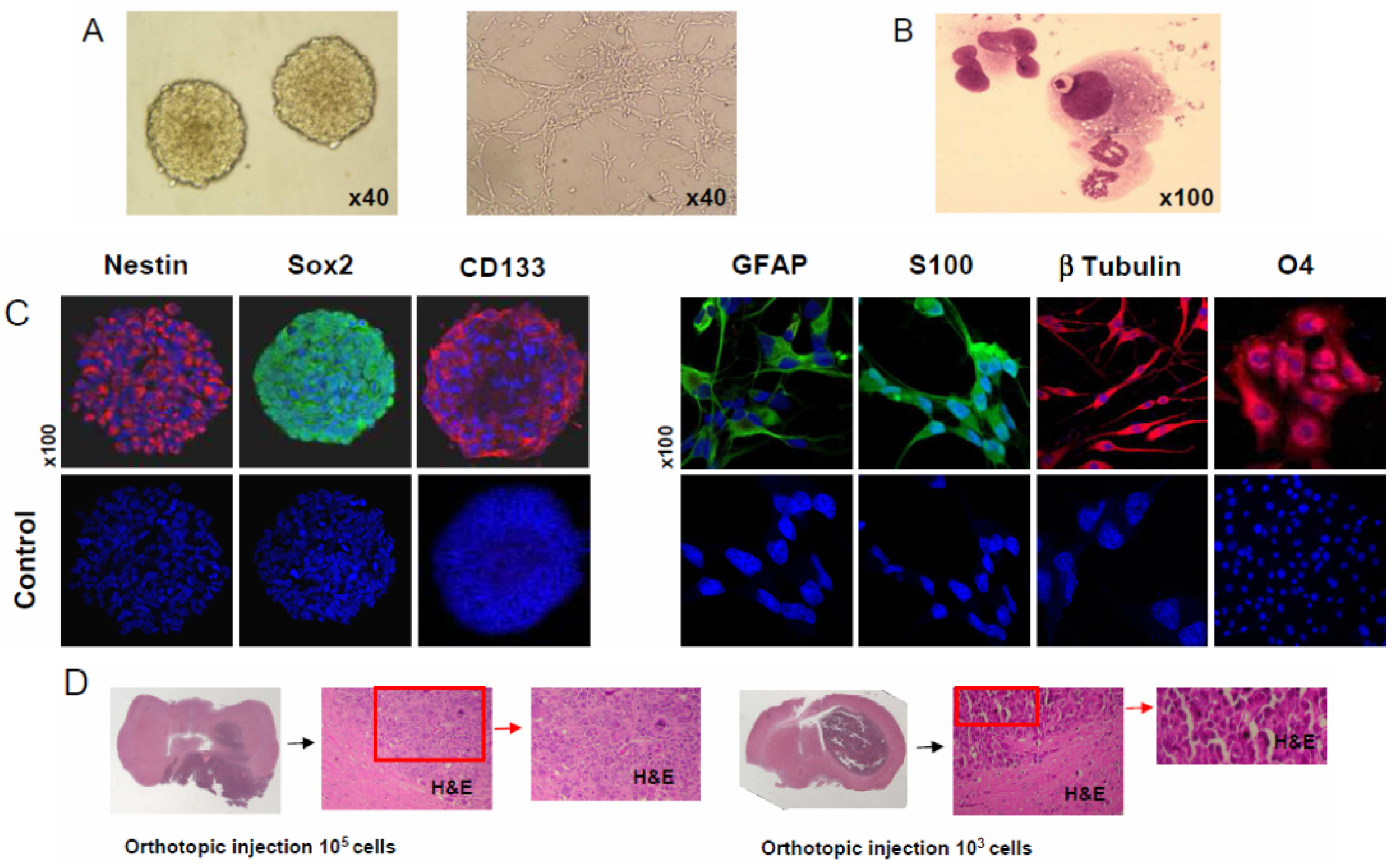

Figure S1. *Cont.*

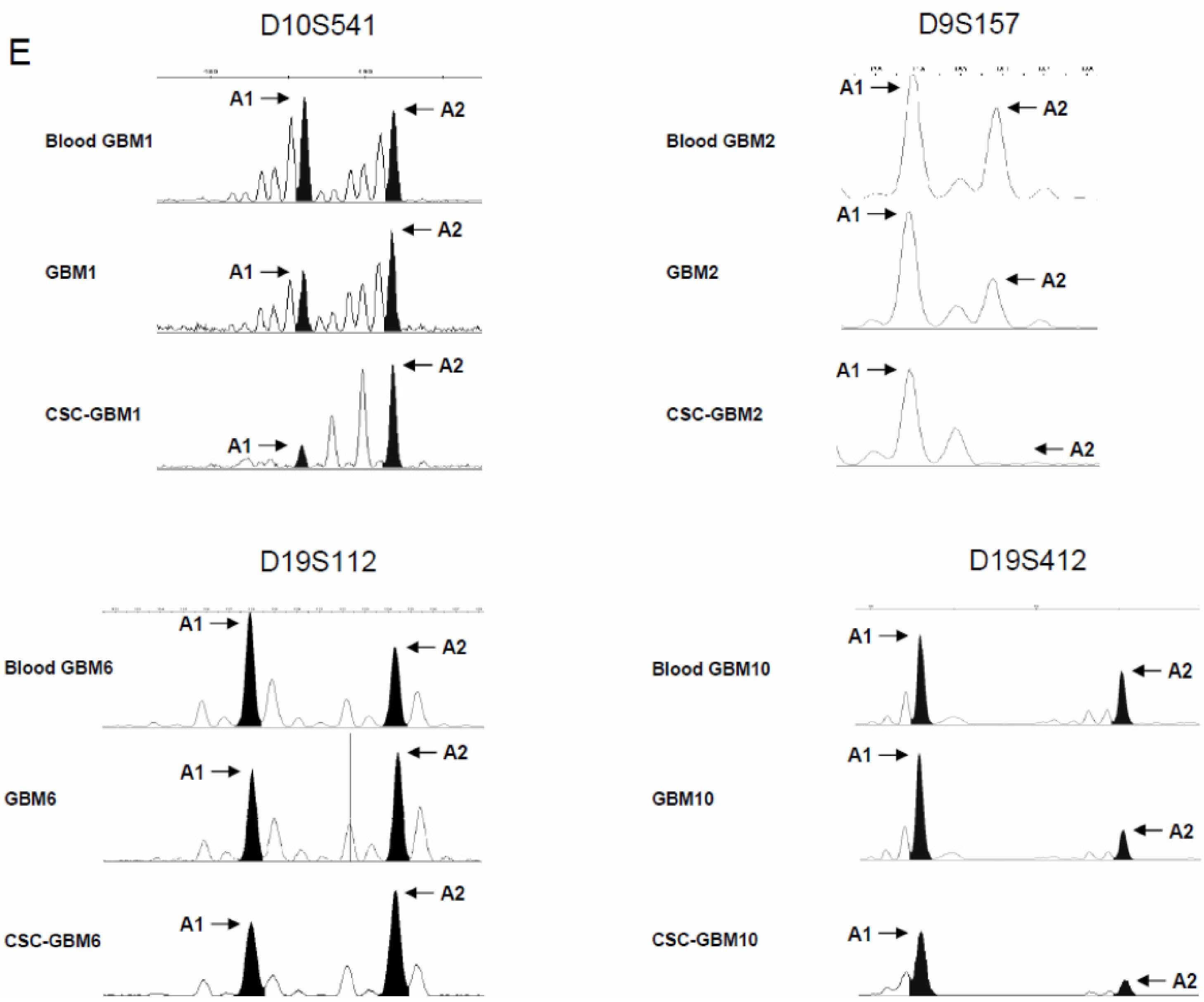

Figure S2: Xenografts from two GBM-derived stem cell lines resembles the original patient tumors. H&E section of xenografts from patient 4 and patient 9 show histological features of GBMs reflecting the patient's original tumor histology. Xenografts and patient's tumor show expression of astrocyte differentiated cell marker (GFAP) and proliferative indices (Ki-67).

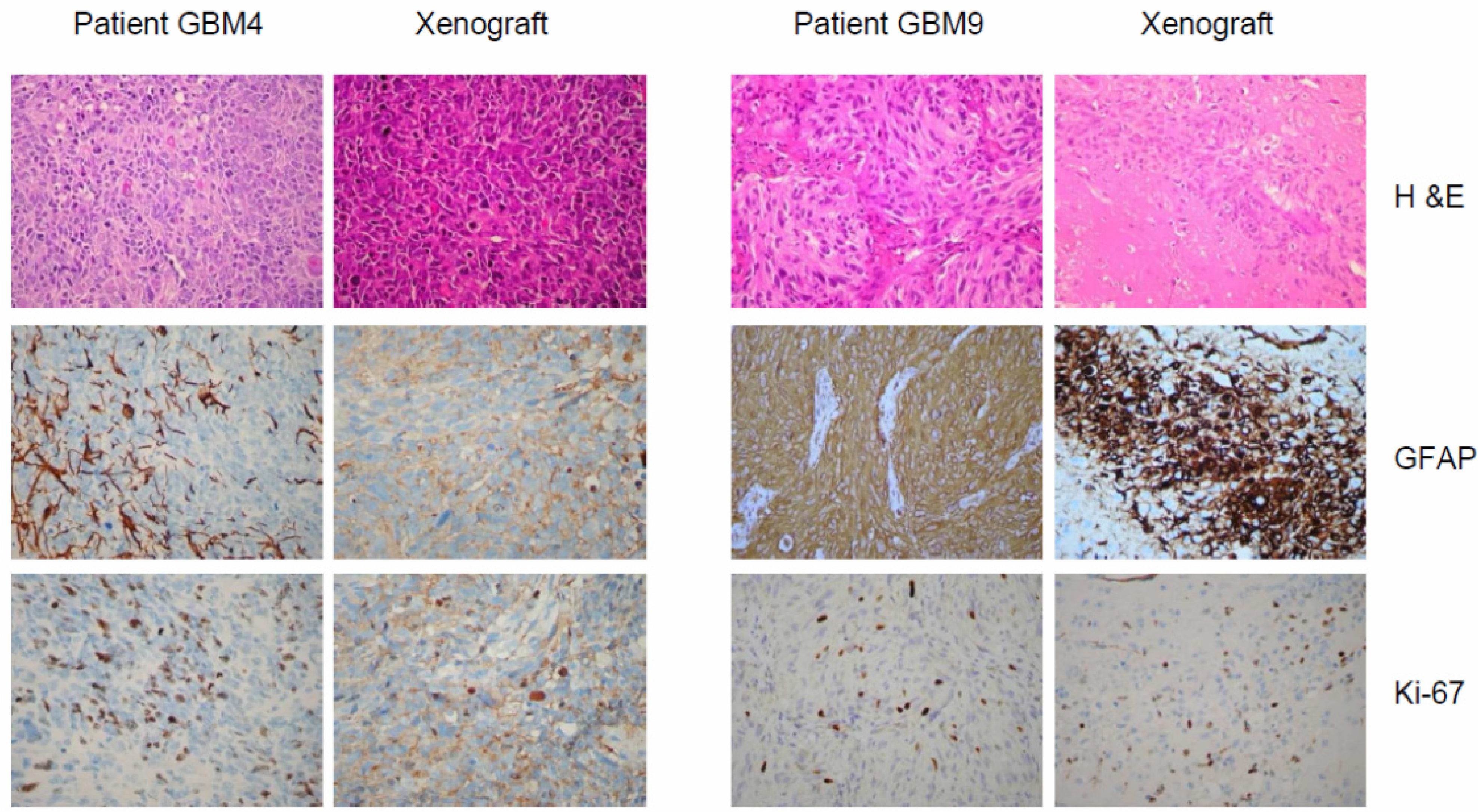

Figure S3: Up-regulation of GFAP in GSCs grown under differentiation-promoting conditions. Representative images of GFAP (green) staining in GBM-10 cell line cultured in serum-free medium or 10% FBS-containing medium. Nuclei were counterstained with DAPI (blue).

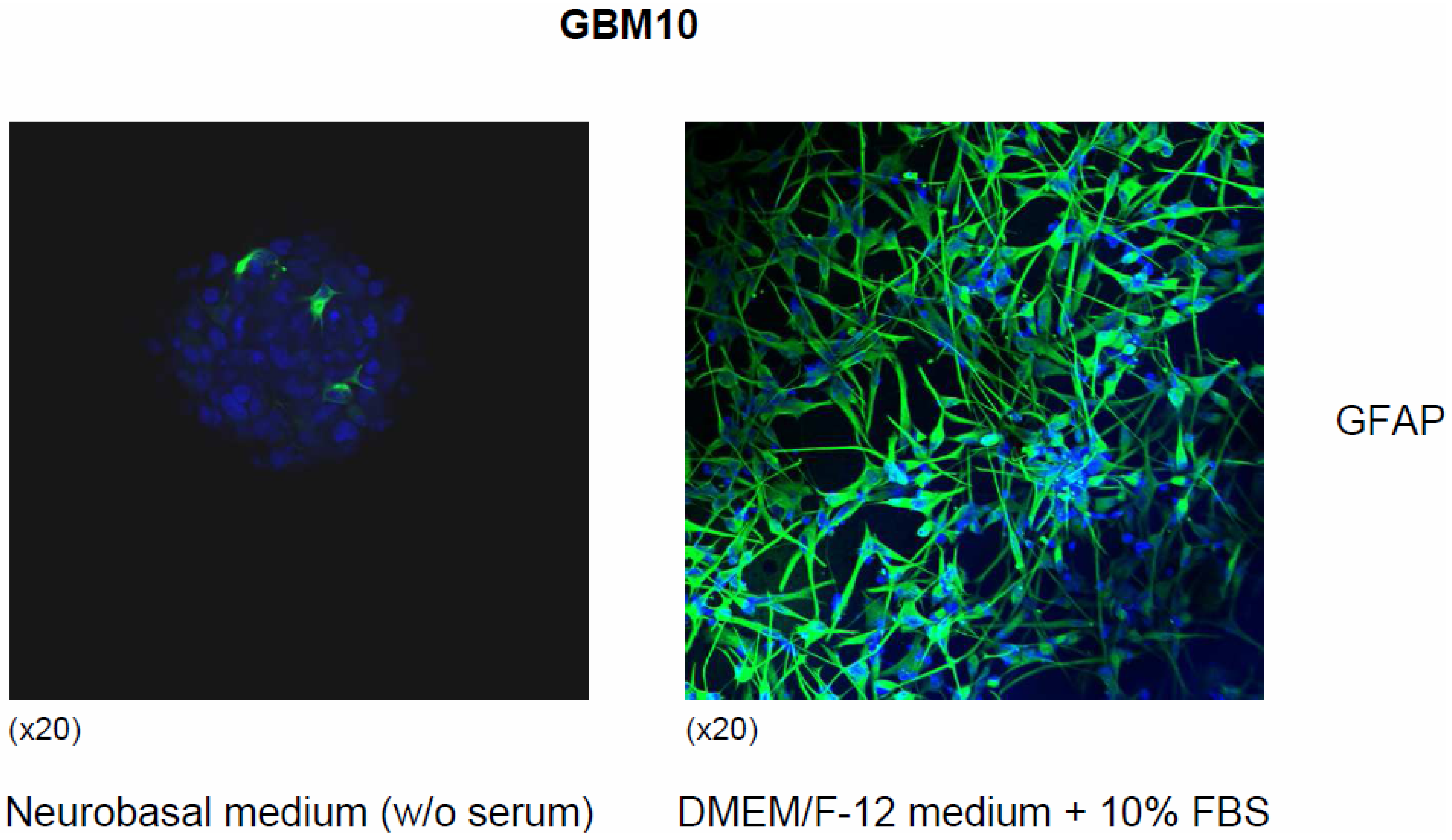

Supplement: Supplementary file 1 [file ijms-13-06983-s001.pdf]
